# The visibility of Eidolon distortions in things and stuff

**DOI:** 10.1167/jov.25.8.12

**Published:** 2025-07-10

**Authors:** Swantje Mahncke, Lina Eicke-Kanani, Ole Fabritz, Thomas S. A. Wallis

**Affiliations:** 1 Centre for Cognitive Science, Institute of Psychology, TU Darmstadt, Germany; 2 Center for Mind, Brain, and Behavior (CMBB), Universities of Marburg, Giessen, and Darmstadt, Germany

**Keywords:** natural image statistics, Eidolon distortions, peripheral vision, image quality, hierarchical modelling

## Abstract

The visibility of alterations to the physical structure of images (distortions) depends on the image content and on viewing conditions. Here we measure human sensitivity to a class of image distortions, Eidolons, applied to image sets containing a range of content, from object images or scenes, to textures and materials. In an odd-one-out task with peripherally presented images, we replicate previous findings that distortions are harder to detect in images which contain large regions of texture or material and fewer segmentable object boundaries. Next, we reason that an image-computable model able to capture the critical aspects of encoding transformations should be able to predict the discriminability of distortion-image pairs, irrespective of image content. We therefore test a variety of image-computable models, treating them as perceptual metrics, using a simple hierarchical regression framework. Of the tested models, the texture statistics of the Portilla and Simoncelli model best predicted performance, beating simple Fourier-spectrum–based transforms and a biologically inspired LGN statistics model. There remains, however, a substantial gap between the best single image-computable metric and an oracle model that has information about the experimental parameters and image labels. This work compliments existing datasets in image distortion discriminability and image quality, and extends existing frameworks for comparatively evaluating the predictive performance of perceptual metrics.

## Introduction

Our environment is filled with different colors, shapes, and textures. With each glance, we see a new arrangement of a visual scene. From these large variations in our environment come many different inputs to our visual system. It remains unclear how such rich visual information is encoded by human perception. One way to approach this question is to measure the discriminability of physical alterations to images, in order to understand which alterations make relatively large (or small) differences to the encoded information available to perception.

### Vision is invariant to some changes in the environment

In general, a distortion can be any physical change to a given image. Although a reduced resolution is one example of a visual distortion, distortions can cover a broader set of changes made to a visual input such as blurring, reduction of color saturation, or addition of Gaussian noise. We here refer to *distortion* as any local or global change to an image that may be applied in gradual levels (zero intensity means no change) and that moves the input away from the original until eventually a different percept is created.

Many distortions of image content are imperceptible. This is particularly evident and practical to study outside the high-resolution fovea, in the visual periphery. Peripheral vision has been hypothesized to mostly be limited by crowding (e.g., Bouma, 1970; Rosenholtz, 2016) and acuity (Rosenholtz, 2016; Strasburger, 2020), and their corresponding changes with eccentricity. In the classical view, the encoding of visual inputs are performed by a set of fixed encoding steps (Rosenholtz, 2016) that proceed in a feedforward, static transformation of the visual input, in correspondence with the known biology at least up to the cortex. However, recent work (e.g., Doerig et al., 2019; Doerig, Schmittwilken, Sayim, Manassi, & Herzog, 2020; Neri, 2017; Wallis et al., 2019) suggests that the information available in the periphery for perceptual decisions can depend on the inputs in ways that the classical view does not predict, or at least underestimates. As an example, sensitivity to the orientation and arrangement of local edge structure can depend on input image properties that are spatially far removed from the edge in question (Doerig et al., 2019; Manassi, Sayim, & Herzog, 2013) or even more semantic in nature (Neri, 2017). Such phenomena are difficult to explain with proximal mechanisms like local contrast gain control or surround modulation, which are typically conceived as operating on more local scales. Therefore, questions arise of how peripheral visual sensitivity can depend on image content, and whether information could be encoded flexibly by the human visual system even at very early stages. Answering these questions would help us to identify what information gets lost and what is preserved in peripheral vision. Studying the visibility of distortions in the periphery is a suitable approach to addressing these questions in the context of photographic images, and one that has been used often in previous work (e.g., Balas, Nakano, & Rosenholtz, 2009; Bex, Solomon, & Dakin, 2009; Bex, 2010; Broderick, Rufo, Winawer, & Simoncelli, 2023; Clarke, Green, & Chantler, 2012; Deza, Jonnalagadda, & Eckstein, 2018; Freeman & Simoncelli, 2011; To, Gilchrist, Troscianko, & Tolhurst, 2011; Valsecchi, Koenderink, van Doorn, & Gegenfurtner, 2018; Wallis & Bex, 2012; Wallis, Bethge, & Wichmann, 2016; Wallis, Dorr, & Bex, 2015; Wallis et al., 2017; Wallis et al., 2019; Ziemba & Simoncelli, 2021).

Here we use Eidolon distortions (Koenderink, Valsecchi, van Doorn, Wagemans, & Gegenfurtner, 2017), which disrupt local image information while keeping pixel value distributions approximately constant. Because a reduced spatial resolution in the periphery alone can not explain peripheral perception, the mapping from visual input onto perceptual representations has to be non-isomorphic in a way that is not just down-sampling the input. We therefore avoid procedures which essentially up- or down-sample an image.

The visibility of distortions is also related to the field of image quality and perceptual metrics. For example, one goal in image compression is to create perceptually lossless compression, which aims to remove as much information as possible from digital images while remaining perceptually indistinguishable from the original image. Because compression schemes create physical changes to the content of images, they can be thought of as a type of distortion. Achieving automated perceptually lossless compression for arbitrary images would likely require an image-computable metric capable of accurately predicting the visibility of a proposed physical change to the image under some assumed task. Earlier research on image quality largely focused on resolution and image compression (e.g., Chandler, 2013; De Ridder, 1998; Wang, Bovik, Sheikh, & Simoncelli, 2004; Zhai & Min, 2020), but has broadened to assess the visibility and suprathreshold similarity of a large variety of distortions (e.g., Hosu, Lin, Sziranyi, & Saupe, 2020; Lin, Hosu, & Saupe, 2019; Piórkowski, Mantiuk, & Siekawa, 2017; Su, Hosu, Lin, Zhang, & Saupe, 2021; Wolski et al., 2018; Wolski et al., 2019; Zhang, Isola, Efros, Shechtman, & Wang, 2018).

### Image categories allow targeted selection of image content

Previous work has shown that the visibility of distortions can highly depend on the image content to which they are applied (Broderick et al., 2023; Wallis et al., 2019). Because it is only feasible to measure distortion sensitivity in a relatively small number of images in our paradigm, we wanted to preselect images to examine the dimension(s) we hypothesize to be relevant, rather than randomly sampling images from a large and diverse dataset. To this end, we oriented our search on the basis of image categories.

The current literature on scene categorization proposes various frameworks to accommodate the notion that visual processing seems to change depending on some features and dimensions. Examples range from high-level categories like things and stuff (Adelson, 2001), indoor versus outdoor (Greene, 2013; Xiao, Ehinger, Hays, Torralba, & Oliva, 2014), man-made versus natural (Greene & Oliva, 2009; Groen, Ghebreab, Prins, Lamme, & Scholte, 2013; Wallis et al., 2019; Xiao et al., 2014), to mid-level representations like scene complexity (Kyle-Davidson, Zhou, Walther, Bors, & Evans, 2023) and number of objects (Groen et al., 2013), down to low-level features based on spatial frequency and contrast (Groen et al., 2013; Seijdel, Jahfari, Groen, & Scholte, 2020). Many of these and similar features have been subject to studies both within the field of visual perception and computer vision (e.g., Caesar, Uijlings, & Ferrari, 2018; Wang et al., 2020; Xiao et al., 2014). Because previous work suggests that the proportion of the image containing texture-like regions is relevant to peripheral perception of certain distortions (Wallis et al., 2019), we wanted to select images for our experiment based on differences in image statistics that are correlated with the relative presence of texture-like content.

### Image statistics as a quantitative measurement of scenes vs textures

Scene categories are most often derived based on the subjective judgments of humans (sometimes participants, and sometimes the experimenters; Greene & Oliva, 2009; Groen et al., 2013; Wallis et al., 2019). This makes sense because categories could be considered to be a cognitive construct, but also creates the complication (for the present study) that judgments about category can differ from person to person. We are interested primarily in scene categories to the extent that they index the proportion of texture-like regions in the image. We, therefore, sought a quantitative measure, computable from the image pixels, to serve as a measure of image features associated with category judgments. Such measures most commonly entail descriptions of features like luminance, contrast, and spatial composition of an image at different scales (Geisler, 2008).

**Figure 1.**
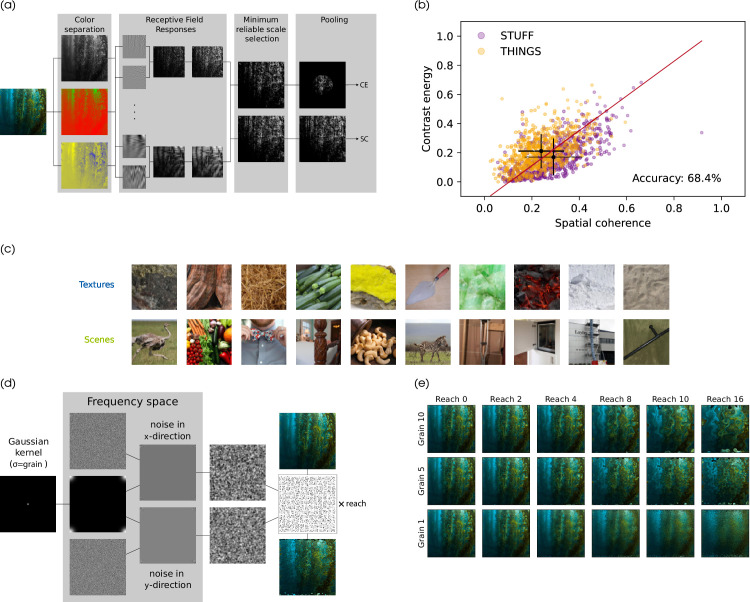
Stimulus selection process. (a) High-level description of LGN statistics algorithm of Groen et al. (2013). (b) SC and CE of 600 THINGS and 600 STUFF images. The accuracy corresponds to the in-sample (training) accuracy of the plotted linear classifier. (c) Example images for the two image types resulting from the linear classifier. (d) Algorithm to produce Eidolon distortions based on Koenderink et al. 2017. (e) Examples for Eidolon-like distortions (Koenderink et al., 2017) by reach and grain.

Groen, Ghebreab, Lamme, and Scholte (2012) introduced two image statistics which they found coincided with images containing more man-made or more natural components. They distinguished a scene’s *spatial coherence* (SC) and its *contrast energy* (CE). These values approximate the scale (CE) and shape (SC) of a Weibull fit to the contrast distribution (Groen et al., 2013). Consequently, low CE (narrow contrast distribution) corresponds with an image whose edges have similar contrast strengths, whereas images with many different contrast values have a higher CE. The SC then mirrors the edge density. The higher the value, the more (high-contrast) edges are present in an image. In comparison natural images showed lower CE and lower SC than images containing more man-made components. When replicating their analysis on images from the THINGS (Hebart et al., 2019) and STUFF (Schmidt, Hebart, Schmid, & Fleming, 2025) database we found a similar shift between the two databases (Figure 1b). Together these results suggest that SC and CE might be informative statistics about image content.

### Our approach

First, we conducted an experiment testing the visibility of Eidolon distortions in different images classified into scenes and textures based on their SC and CE (see Experiments). We used these image statistics to classify images into these image types in a more reproducible, quantitative fashion than used in previous work (Wallis et al., 2019). Based on the results of Wallis et al. (2019) and Valsecchi et al. (2018), we expected that Eidolon distortions would be harder to discriminate from the undistorted original in images that contain more texture compared to images that contain little texture.

Second, we used these data to test the degree to which a selection of fixed encoding models, which use specific image features considered to be represented early in the visual processing stream, could predict discrimination performance (see Metric-based modelling). To this end, we treated fixed encoding models as perceptual metrics. We computed the difference between each pair of distorted and undistorted images according to the metric, then used a simple regression framework to compare the predictive power of each metric to an oracle model that used information on the experimental parameters themselves. In this way, we quantify both the relative predictive usefulness of different image-computable metrics, and also estimate a lower bound on how well a new metric could potentially predict performance.

## Experiments

### Methods

#### Participants

In the pilot experiment, two of the authors participated. For the main experiment participants were recruited from the student body of TU Darmstadt and received course credits for their participation. In total 32 participants (62.5% female, ages between 19 and 33 years) took part in both experiments. All participants were asked to wear their normal optical correction if necessary, but we did not measure visual acuity or contrast sensitivity. All participants provided informed consent and could withdraw from the study at any time. The procedures adhered to the Standard 8 of the American Psychological Association’s “Ethical Principles of Psychologists and Code of Conduct” (2017), and were approved by the Technical University of Darmstadt Ethics Commission (Application number EK 77/2022).

#### Stimuli

##### Image selection.

We selected all images in both experiments from the THINGS (Hebart et al., 2019) and STUFF image databases (Schmidt et al., 2025) scaled to 350 px in width and height. To arrive at equal set sizes and avoid bias we randomly selected 600 images from the THINGS database together with the 600 images from the STUFF database for the initial analysis.

Preceding the experiment we analysed the images using an LGN-inspired algorithm (Figure 1a) based on the work of Groen et al. (2013) which produces two characteristic local contrast values per image: SC and CE. The computation of these values are described in detail in the Appendix.

Based on the results (Figure 1b), we selected images for two conditions. The first condition, which we refer to as *scenes*, has high CE and high SC. The second condition, *textures*, has low CE and low SC. In the pilot experiment, we randomly selected 20 images for each condition characterized by the upper left and lower right quadrant of Figure 1b. For the main experiment, we extended the dataset to a total of 60 images per image condition. Using a linear support vector machine based on CE and SC (in-sample accuracy: 68.4%) we categorized the images into the two image conditions and chose random images from the resulting sets. Figure 1c shows an exemplary sample for images from the two image types.

##### Distortions.

For distorting the images, we chose a variation of the Eidolon distortions introduced by Koenderink et al. (2017). With this model, a distortion is applied to an image by disarraying its pixels according to a Gaussian random vector field (Figure 1d).^1^ The distortion is varied through two parameters *reach* and *grain*. While the spatial frequency of the distortion scales inversely to the grain parameter, the reach encodes the intensity of the distortion. The original Eidolon factory additionally includes a scale decomposition using a coherence parameter, which governs the correlation of disarray between scales. Here we chose not to apply any scale decomposition, leaving us with only the two parameters, grain and reach. Examples of distortions for different parameter values are shown in Figure 1e. The computational steps following the procedure in the Eidolon factory (Koenderink et al., 2017) is given in Appendix.

In the pilot experiment, we considered the grain values 1, 3, 5 and 10. Each participant (in this case two authors) chose different reach values independently to effectively measure the proportion of correct responses in difficult trials and constrain the psychometric function at the same time.

For the main experiment, we included grain 7 as an additional parameter value. For each participant and each grain, we measured the distortion visibility at reach levels 1, 2, 3, 4, and 8. To reduce noise in the single participant data, we measured additional reach levels for certain grain values if necessary.

#### Setup

We implemented the experiments with the use of PsychoPy 2023.1.3 (Peirce et al., 2019) under Python 3.8.18. Distortions were created with Numpy 1.23.5 (Harris et al., 2020), OpenCV-python 4.8.1.78 (Bradski, 2000), Pillow 10.0.1 (Lundh & Clark, 2015), SciPy 1.10.1 (Virtanen et al., 2020), Pandas 2.0.3 (McKinney, 2010; The Pandas development team, 2020), and scikit-image 0.21.0 (van der Walt et al., 2014). The experiment was performed on a PROPixx projector system (VPIXX Inc., Saint-Bruno, QC, Canada) with a resolution of 1,920 px × 1,080 px and a refresh rate of 120 Hz controlled by a Linux computer (Ubuntu 22.04 LTS) via a DATAPixx3. Responses were given through button presses on a standard keyboard.

#### Procedure

**Figure 2.**
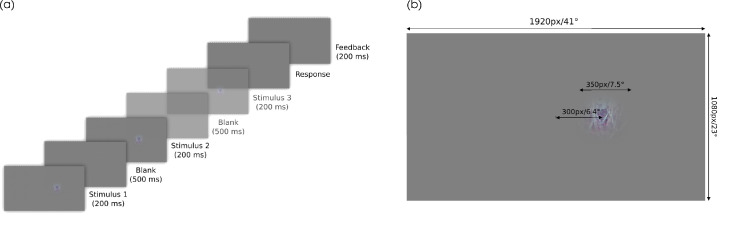
Experimental procedure. (a) Time course of one experimental trial. In the pilot experiment only two stimuli were presented (two-interval forced choice). In the main experiment, a third stimulus with inter stimulus blank screen was shown (transparent frames; odd-one-out). (b) Spatial arrangement of each trial.

The participants were seated 1.3 m from the screen. The stimuli were presented 6.4° right of fixation and spanned a 7.5° of visual angle in diameter. They were blended into the gray background with a Gaussian mask (*SD* = 1.25°) (Figure 2b).

##### Pilot experiment.

The participants performed a two-interval forced choice task in each trial. In each trial, they were presented with the same source image, once unaltered and once distorted (with order randomly determined). They then were asked to identify the distorted image and indicate their decision by a button press. The temporal arrangement of a single trial is shown in Figure 2a.

Participants performed multiple trials as blocks of varying length. Each block contained stimuli with a fixed distortion grain and from a fixed image condition (texture or scene). The measured reaches were varied both within a block and between the blocks. All 20 images from a given image type were presented the same number of times per block, ranging between one and four times for each condition. The order of images and reach values within a block were randomized.

##### Main experiment.

Because participants of the pilot experiment reported that it was sometimes hard to identify which image was the original even though they were able to tell that there was a difference, we changed the task to an odd-one-out task for the main experiment. The spatial layout in each trial was not changed, that is, each stimulus was presented in the same manner as described at the beginning of this section.

For the oddity task, the participants sequentially saw three stimuli, of which two were identical and one was different. The two stimuli always were one image and a distorted version of the same image. The target stimulus could be either the distorted or the original image. The target appeared randomly in the first, second, or third interval. The participants indicated by button press which interval contained the odd stimulus. (Figure 2a). Note that it is possible to perform this task without identifying which of the images are distorted, because participants only need to detect which image is different. Therefore, the main experiment only measures sensitivity to the change in the image introduced by the distortion, and cannot be used to infer what the distortions looked like to participants (i.e., appearance).

The trials were again shown in blocks with a fixed distortion grain. Every participant conducted the blocks for a subset of 10 images for each image type, which was the same subset across all blocks of that participant. Each image per reach value was presented once within a block. Experimental conditions were presented randomly on each trial.

Preceding the experimental blocks participants did a set of practice trials at a mid-level grain and one high-reach value. At the start of the practice block the participants were shown all 20 images they would see during the experiment for familiarization.

#### Data analysis

We conducted the data analysis using Python 3.11.5 with the packages Pandas 2.2.2 (McKinney, 2010; The Pandas development team, 2020), Numpy 1.24.3 (Harris et al., 2020), Psignifit 0.1 (Schütt, Harmeling, Macke, & Wichmann, 2016), Numpyro 0.15.0 (Bingham et al., 2019; Phan, Pradhan, & Jankowiak, 2019), Jax 0.4.30 (Bradbury et al., 2018), Pillow 10.3.0 (Lundh & Clark, 2015), SciPy 1.11.3 (Virtanen et al., 2020), scikit-image 0.22.0 (van der Walt et al., 2014), PyTorch 2.2.0 (Ansel et al., 2024), and Plenoptic 1.0.2 (Duong et al., 2023). All plots were generated using Matplotlib 3.8.0 (Hunter, 2007).

##### Preprocessing.

We realized that some participants’ response accuracy did not improve with higher reach (intensity). Therefore, before conducting the subsequent analysis we determined the overall performance in percent correct for each participant. Participants who had a performance below 60% correct over all trials were excluded from the analysis, because we expect these participants did not understand the task correctly. This resulted in the exclusion of 4 participants, leaving 26 participants for data analysis. The data for these four excluded participants are shown in Appendix.

##### Modelling.

**Figure 3.**
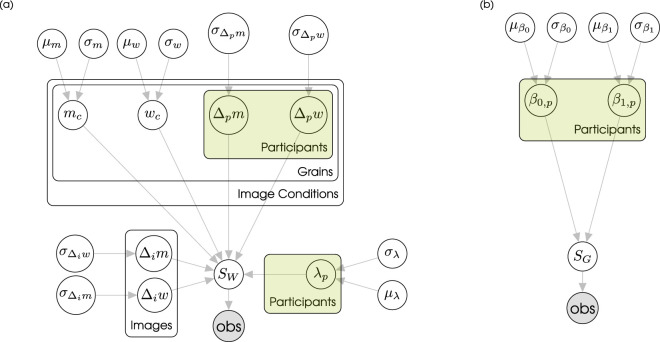
Model graphs for (a) the experiment-based model and (b) the model based on a perceptual metric. In the experiment-based model, outputs are generated through a compressed Weibull function with width *w*, threshold *m*, and lapse rate λ. Threshold and width are given through normally distributed values for each experimental condition plus a zero-mean offset (Δ) for the participant and specific image. The metric based model applies a cumulative Gaussian fit on a linear Regression with normally distributed β-weights per participant.

We fitted psychometric functions to the proportion correct performance using a hierarchical model assuming binomial errors (Figure 3a) and employed Bayesian inference to estimate the posterior distribution for the model parameters.

The probability of a correct response was modelled as a Weibull distribution with threshold *m* and width *w* following the parametrization of Schütt et al. (2016, Table A.1):

$$\begin{array}{ccc} F_{W}\left(x;m,w\right) & \;= & 1-\exp\left(\log\left(0.5\right)\cdot e^{c\cdot \frac{\log(x)-m}{w}}\right),\\ S_{W}\left(x;m,w,\lambda \right) & \;= & \gamma +\left(1-\gamma -\lambda \right)\cdot F_{W}\left(x;m,w\right), \end{array}$$

where γ is the chance level performance (Pilot experiment: $\gamma =\frac{1}{2}$, Main experiment: $\gamma =\frac{1}{3}$), λ denotes the lapse rate and *x* is the stimulus intensity in reach values. The observed responses were then considered to arise from a Binomial distribution with probability of success given by *S*_*W*_.

This model has three main parameters: *m*, *w*, and λ. We modelled the random effects of the participant (*p*) and the specific image (*i*) as an offset to the condition-wise threshold (*m*_*c*_) and width (*w*_*c*_):

$$\begin{array}{ccc} m & \;= & m_{c}+\Delta_{p}m+\Delta_{i}m\\ w & \;= & w_{c}+\Delta_{p}w+\Delta_{i}w. \end{array}$$

The condition-wise mean parameter of *m*_*c*_ and *w*_*c*_ were given normally distributed priors of μ∼N(1,1). Each offset had a zero-mean normal prior. The priors for all variances were set to

$$\begin{array}{c} \sigma ∼\log N(-1,1). \end{array}$$

A priori, we strongly expect lapse rates λ to be close to zero. We therefore chose a beta-distribution as prior for the mean λ and truncated the normal distribution for each participant’s lapse rate between 0.0 and 0.2:

$$\begin{array}{ccc} \mu_{\lambda } & \;∼ & \text{Beta}(1,10),\\ \lambda_{p} & \;∼ & N(\mu_{\lambda },\sigma_{\lambda };>0.0,<0.2). \end{array}$$

### Results

**Figure 4.**
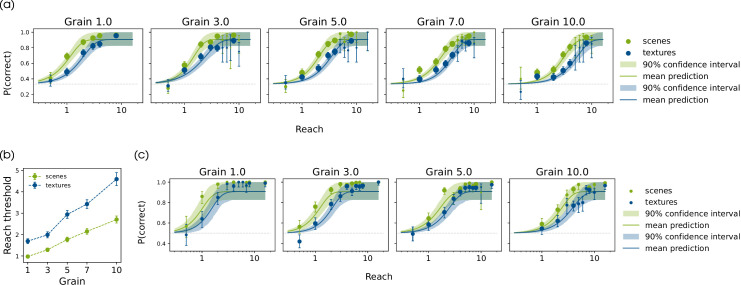
Psychometric function fits for collected data. Data points show the mean performance for the data pooled over all participants. The size of the data points indicate the amount of pooled data, that is, the larger the point the more data is included. Error bars on the data points show the 90% credible interval of the beta distribution estimate. (a) Fitted functions for data of the main experiment by image condition and grain. (b) Posterior estimate for thresholds in the main experiment by grain and image condition. Error bars indicate the standard deviation of the posterior distribution. (c) Fitted functions for data in pilot experiment by image condition, and grain.

Over all conditions and experiments, we find that the proportion correct increases depending on distortion reach (Figure 4). Hence, reach can be considered a valid distortion intensity or difficulty measure.

#### Distortion changes are harder to detect in textures

In both experiments, we see a shift of the psychometric function between scenes and textures (Figures 4a, 4c). In particular for the main experiment (Figure 4a) we can see clear results, as the credible intervals are narrower and only overlap at the tails of the function. This indicates that the changes produced by the distortions are easier to detect in scenes than in textures for all grains. Additionally, Figure 4b shows that the detection threshold increases for textures compared to scenes, indicating that the detection of the change was harder for the textures. For all grains, the 95% highest posterior density interval (HPDI) on the threshold of the difference distribution between scenes and textures does not include zero (HPDI_95%_ ∈ [0.340, 2.718] for all grains), that is, there is a credible difference between the thresholds.

#### Changes with higher distortion grain are harder to detect

Overall, performance decreases if grain increases, indicating that the task is more difficult at higher grains. This effect can in particular be seen in Figure 4b, as the thresholds increase for both image conditions with larger grains. For both image conditions, the threshold increase is significant with each grain step (HDPI_95%_ ∈ [0.028, 1.670] for both image conditions, and each pairwise difference). Note, that perceptually the appearance of a high grain distortion exhibits bigger blob-like structures with a low frequency. Hence changes from distortions with lower spatial frequency are harder to detect. (Presumably the full function is in reality non-monotonic, since we would expect very low grains to cause distortions that fall outside the range of the acuity threshold, implying that reach thresholds should get larger as grain approaches zero.)

## Metric-based modelling

The model fit to the data in the previous section (Figure 3b) uses information about the parameters of the distortions we applied (reach, grain) to predict performance. In addition, it allows the effect of reach and grain to vary depending on which image and which participant the response in question arises from; this variance is quantified in a data-driven way (i. e. via the posterior distribution). However, the model does not depend directly on the content of the images (only their labels). Therefore, while the experiment-based model provides a useful description of performance variance in the data, it does not allow us to associate performance changes with sets of image features themselves.

To understand how image information is encoded by the visual system, we would like to be able to describe what information might be available using an image-computable model. Such a model could also describe how the perceptually relevant information changes as images are distorted. Applying this logic, we can compute candidate image-dependent model responses, then define difference measures, which we refer to as metrics,^2^ that give us a measure for how perceptually different the distorted image is from the original based on the corresponding statistic. An image-computable metric that captures the visibility of image changes should then correlate highly with human performance across conditions, and therefore show good performance in predicting which particular images and corresponding distortions are relatively easy or difficult to discriminate.

To illustrate this approach, here we choose a relatively small set of candidate image features or image-computable models (Table 1). We consider simple differences in color contrast, frequency spectrum differences, the aforementioned CE and SC metrics of Groen et al. (2013), and the popular Portilla and Simoncelli (2000) texture statistics. Finally, we also include the more recent human visual system inspired image quality metric FovVideoVDP (Mantiuk et al., 2021), which instantiates many classical mechanisms of human vision (such as channel decomposition, contrast sensitivity weighting and cross-channel masking) into an image-computable model that yields a difference map and a just-objectionable difference (JOD) score. Details on the calculation of these metrics are provided in the Appendix.

**Table 1.** Image-computable analysis metrics. All metrics apart from the MSE, JOD, and cosine similarity were considered as a direct difference between the values of original and distorted image as well as a relative difference which was the direct difference normalised by the statistical value of the original image.

Image information	Metrics
Pixel values	Mean squared difference
Red-green mean	(Normalized) difference
Yellow-Blue mean	(Normalized) difference
Frequency spectrum	(Normalized) Manhattan metric
Fourier intercept (*FI*)	(Normalized) difference
Fourier slope (*FS*)	(Normalized) difference
LGN statistics (Groen et al., 2013)	(Normalized) Euclidean distance
Contrast energy (*CE*)	(Normalized) difference
Spatial coherence (*SC*)	(Normalized) difference
Portilla and Simoncelli (2000)	(Normalized) Euclidean distance and cosine similarity
FovVideoVDP (Mantiuk et al., 2021)	JOD

We first analyze how the Eidolon distortions change these metrics. Second, we treat the differences between unaltered and distorted images produced by the metrics as difficulty measures, then use these differences as features in simple hierarchical regression models that only depend on the metric and the participant (Figure 3b). In other words, we assess the extent to which the metrics can predict the visibility of a particular distortion applied to a particular image, while allowing the association of each metric’s prediction performance to be different for each participant. This approach extends previous research used to quantify performance of image quality metrics using regression models (e.g., Seshadrinathan et al., 2009; To, Lovell, Troscianko, & Tolhurst, 2010; Wang et al., 2004; see also Mikhailiuk, Perez-Ortiz, & Mantiuk 2018; Perez-Ortiz et al. 2020).

To then test the predictive power of each metric, we compare how well the models fit the data by means of model comparison. The metric with the most predictive power is then the one for which the fitted model performs best in our model comparison. Here, the previous experiment-based model serves as an oracle model that has all the information about the experiment. As such the experiment-based model provides us with a maximal possible performance when including all information about the experiment.

### Methods

#### Distortion analysis

We used different image information mainly in form of natural image statistics to measure the changes which distorting the image results in. The considered image information is summarized in Table 1. Based on the different information we computed suitable difference measures to quantify the effect of the distortion on the images. The computation of each metric is described in Appendix.

#### Metric-based models

Based on the results of the distortion analysis we defined various difficulty measures (Table 1) and analyzed the observer’s performance in the main experiment dependent on these metrics.

For the majority of metrics, a value of zero corresponds to no difference between the two images. Since the cosine similarity produces values between zero and one with one being no difference, we adapted these values by shifting them by one, such that zero represents no difference here as well. Similarly, for the quality of FovVideoVDP (Mantiuk et al., 2021), in JOD the maximum of 10 represents equal images. Hence here again we shifted the values by 10 to obtain equality at 0.

We again defined a hierarchical model only taking into account the perceptual metric and interindividual differences (Figure 3b).

As a predictive function for the metric-based model we used a linear Regression with a cumulative standard normal distribution:

$$\begin{array}{c} S_{G}\left(x;\beta_{0},\beta_{1}\right)=\gamma +\left(1-\gamma -\lambda \right)\cdot F_{N}\left(\beta_{0}+\beta_{1}\cdot x\right), \end{array}$$

with chance-level performance $\gamma =\frac{1}{3}$, lapse rate λ = 0, cumulative Gaussian *F*_*N*_, and intensity *x*. We choose to remove the lapse rate here, since otherwise if we have a metric with no predictive power, for example, constant performance, the data could be fitted with a very large λ such that the relevant parameters (the β-weights) lose their intended meaning (the parameters become unidentifiable).

To capture varibility between participants while allowing for partial pooling, we model each participant’s regression weights β_*i*, *p*_ in a multilevel model (i.e., participant is a random effect), where

$$\begin{array}{c} \beta_{i,p}∼N\left(\mu_{\beta_{i}},\sigma_{\beta_{i}}\right),\;i\in \left\{0,1\right\}. \end{array}$$

The priors for the population-level hyperparameters were set as zero-mean Gaussians ($\mu_{\beta_{0}}∼N\left(0,2\right)$, $\mu_{\beta_{1}}∼N\left(0,1\right)$ and σ∼logN(0,1)).

#### Model comparison

Because some metrics only produce non-negative differences between the images, for example, the MSE, and others do not, we considered the absolute values for all parameters. Additionally, we applied a logarithm to each metric to arrive at data similar to a sigmoid function. Because the metrics varied greatly in their numerical span, as a final step before model comparison we transformed each metric into a *z*-score. In result we fitted a total of 52 models to the experimental data.

We reduced the number of models by running a pairwise comparison between the absolute change and the relative (normalised) change version for all parameters where these two versions exist. For the final main comparison for each metric only the better one was considered. This process resulted in 13 models for the final model comparison, one for each metric plus the experiment-based model.

We then compare these models on their predictive performance on unseen data. To reduce computational requirements we use an approximation to leave-one-out crossvalidation on the expected log predictive density (ELPD) scale (the WAIC; Vehtari, Gelman, & Gabry, 2017; Watanabe, 2010), to compare each metric-based model with the experiment-based model.

### Results

**Figure 5.**
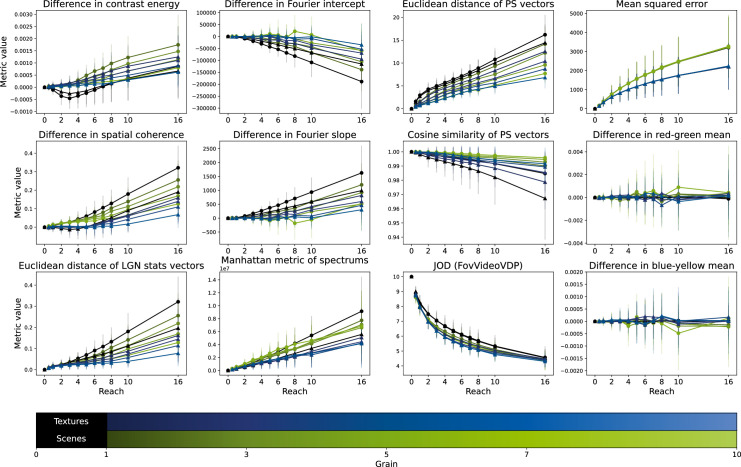
Change in statistics owing to distortions. Each panel shows the average metric value over the images used in the experiment dependent on distortion reach, grain, and image condition. Image conditions are depicted with both color and shape differences, with Textures in blue triangles and Scenes in green circles. Error bars show the standard deviation. All difference scores are expressed as original - distorted, so that positive values mean a higher absolute value in the original image.

Figure 5 shows the average metric values over the image used in the main experiment for each metric we considered dependent on the distortion parameters grain and reach and grouped by the image condition.

#### Metrics with spatial difference information capture the distortion intensity

We can observe that on average every metric apart from the difference in color means changes more with larger reach values. This indicates that the larger the distortion the further apart the original and the distorted image are as measured by the metrics. Hence, all these metrics should be able to encode the intensity of the distortion. Notably, an increase in the difference in SC and the Euclidean distance of the LGN vector can only be seen for fairly large reach values.

#### Distortion grain affects image statistics differently

All statistics that include spatial frequency information in some form show a dependence on distortion grain. The only metric for which we cannot observe any dependence on grain is the mean squared error which has the same curves for each grain. This is likely due to the fact that the MSE has no means to measure spatial frequency and therefore all distortion grains produce very similar MSE values. Although there seems to be a slight dependence of the color change on grain, there is no obvious regularity in this dependence. For the majority of the remaining statistics (all but JOD, CE, and power spectrum) the larger the grain the smaller the change in the statistical value, that is, the metrics are more sensible to distortions with high spatial frequencies. Interestingly for the JOD we observe a dependence on grain inverse to the other metrics, that is, quality measured by JOD decreases more for larger grains. The difference in CE shows a similar pattern of decreasing difference for larger grains; however, there is an exception to this rule as for grain one the difference is only larger than the rest for small reach values, and in that interval also negative contrary to the remaining more intense distortions. This outlier position remains when considering the absolute value of the differences. Note that, for the smallest grain, the edges in an image get disrupted such that they may no longer appear as edges except at low spatial frequencies, whereas for larger grain values the edges appear more curved rather than disrupted, only being notably disrupted for larger reach values, which then produces an increase in CE. Last, the change in the power spectrum shows a notably different dependence on grain as for small reach values large grains produce a larger change than small grains.

#### Image condition interacts with the effect of distortion

Again, for mean color changes the image condition has no obvious effect. Similarly, the JOD only shows very small dependence on the image condition with scenes producing slightly higher values, that is, less difference from the original, than textures for large grains. We can observe the same shift direction for the cosine similarity of the Portilla and Simoncelli (2000) vector, as the curves for scenes are compressed toward one, which would be no difference. For all other metrics, there is a clear systematic shift between scenes and textures, such that for all distortions scenes produce larger metric values than textures, that is, the metrics are more sensitive to changes in scenes than to those in textures.

#### Unnormalized metrics produce better fits

The pairwise model comparison between the absolute stimulus differences and differences normalized by the original image value (conducted for all metrics except cosine similarity, MSE, and JOD) revealed the unnormalised metrics to better fit the data for all metrics except the difference in SC and the Euclidean distance of LGN vectors. This indicates that for the majority of measurements the absolute size of the change is a better predictor of the difficulty than the proportional change.

#### All metrics explain some variance in the perceptual data

**Figure 6.**
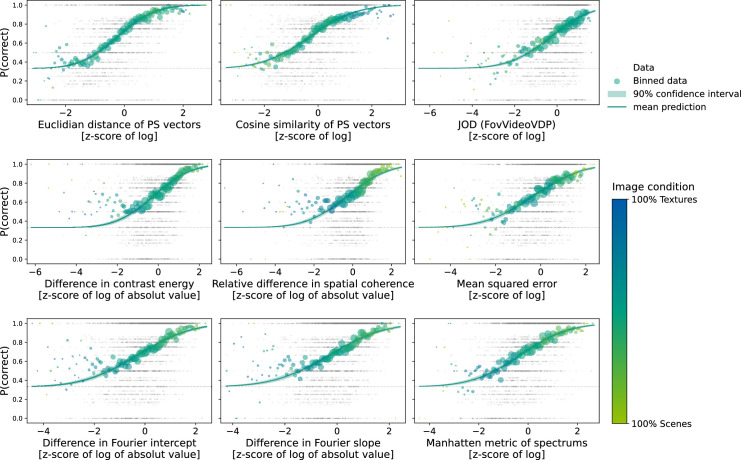
Model fits for different perceptual metrics. Each plot contains the performance data of all participants in all trials of the main experiment dependent on the difference between the distorted image to the original in that trial as measured by the specific metric. The gray data points show the individual Bernoulli trials. For better visualization we binned the data. For the binned data point the size indicates the number of trials pooled in this data point and the color indicates the proportion of scenes or textures in the pooled data.

Figure 6 shows the fitted psychometric functions based on the hierarchical regression model for a subset of the metrics we analyzed. For all metrics, we can see a sigmoid shape of the binned performance distribution such that better performance coincides with larger metric values. Hence, all considered metrics are suitable to fit the performance data to some degree. However, the asymmetry and scatteredness of the data for some of the metrics, suggest that the collected data might not be sufficient to fit good models for each metric.

#### Most metrics do not differentiate the image condition

The color of the binned data in Figure 6 indicates the ratio of scenes to textures in the data pool. For the majority of metrics, there is a slight shift toward the tails such that higher metric and better performance values contain more scenes while the lower end contains more textures. Similar to the results of the distortion analysis the shift is inverse for the Cosine similarity of the Portilla and Simoncelli (2000) vectors. The JOD and Euclidean distance of the PS vectors have more or less the same ratio overall metric values, mirroring the small dependence of the change through distortion on image condition for these metrics.

#### Model comparison

**Figure 7.**
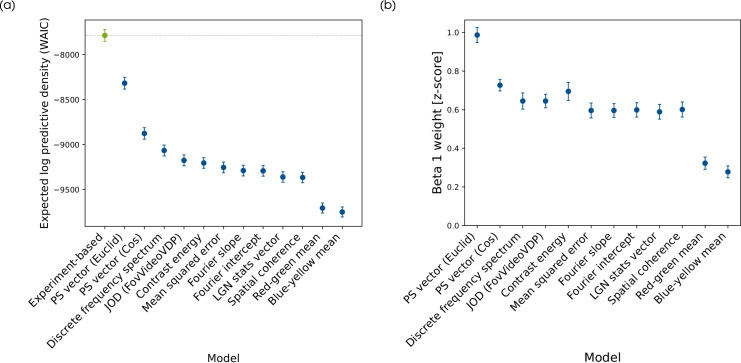
Results of model comparison. (a) ELPD as result of pairwise comparisons between the experiment-based model (green) and each metric-based model (blue). Error bars indicate the standard error of the ELPD estimate. Dashed line shows mean value for experiment-based model for comparison. (b) Estimations and 90% credible interval of the β_1_-weight in the metric based models sorted by the models performance in the model comparison.

The results of the model comparison are shown in Figure 7a. Here higher ELPD values indicate a better prediction of out-of-sample data.

The experiment-based model which includes image and participant-level random effects can predict the data best. Of all the considered metrics, the models based on differences in the Portilla and Simoncelli (2000) vectors predict the data best, in particular, the Euclidean distance of these vectors comes somewhat close to the prediction of the experiment-based model. The β_1_ weight being close to one supports the relevance of the Euclidean distance in PS vectors for human performance prediction (Figure 7b).

In accordance with the results of the distortion analysis, shifts in mean colour predict the data worst. The low relevance of mean colour shift for the human performance in the studied task is also evident in the low β_1_-weight in these models (Figure 7b).

The remaining models vary only slightly in their prediction performance. In particular, the mean squared error which showed no dependence on grain still predicts the data as well as the models including spatial frequency information. Interestingly, combining the information of CE and SC into one metric with the Euclidean distance of the LGN stats vector does not improve the model predict. Comparing the full spectrums instead of only considering the Fourier slope or intercept increases the predict slightly.

The β_1_-weights mostly mirror the results of the model comparison, that is, better predicting models have a higher weight on the metric. An outlier here is the CE, which does not follow the course of the ELPD values, that is, the weight of the CE in the model is higher than the model comparison would suggest.

## Discussion

**Figure 8.**
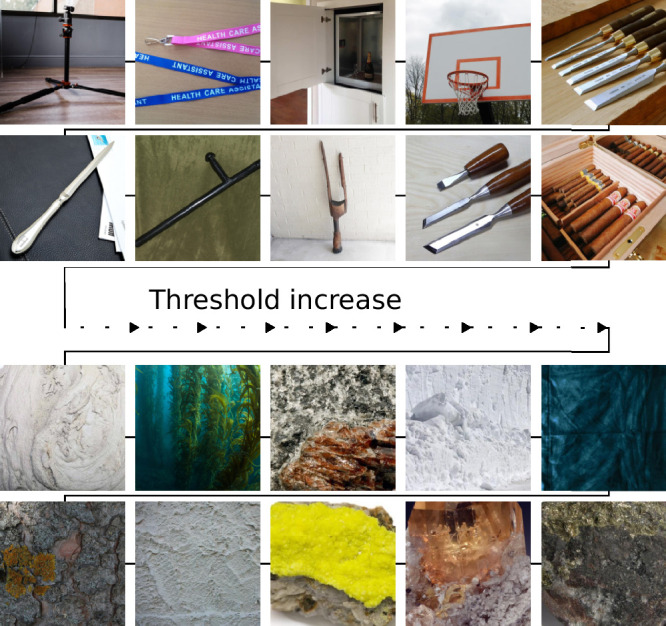
Easiest (top) and hardest (bottom) images in the experiment, sorted by threshold.

In this work, we extend previous regression frameworks for analysing the connection between image statistics and behavioural data. Through analysis of distorted images, we found that a variety of image-computable difference measures can to some extent quantify the perceptual changes induced by the Eidolon distortion. Extending the results of Wallis et al. (2019) and Broderick et al. (2023), our experimental data support the notion of image-content-dependent visibility of Eidolon distortions in colored images, such that the changes they introduce are harder to detect in images containing a lot of texture. Visual inspection of the easiest and hardest images from the experiment (Figure 8) further supports this idea. In particular, we found that distortions of low spatial frequency (larger grain), are particularly hard to discriminate as the perception threshold increases with grain (Figure 4b). Although in the present study we did not use eyetracking to monitor fixations, we think it is unlikely that participants systematically diverged from central fixation, because the psychophysical results qualitatively replicate those of Wallis et al. (2019), where eyetracking was used to control presentation location.

The modelling indicates that all metrics containing some information on the spatial change can predict the discrimination data to some extent. The Portilla and Simoncelli (2000) texture parameters perform best of the models we consider here in predicting human discrimination performance for distortions. Finally, we reimplemented the SC and CE statistics of Groen et al. (2013), and found a similar shift in these LGN statistics between things and stuff to what was previously described in their work between man-made and natural scenes.

###  

#### Quantifying the perceptual difference between scenes and textures

The results presented here provide information regarding which image features are important to include in a future fixed-encoding perceptual model. The low performance of the LGN statistics in the model comparison suggests that these might not contain enough information to derive the perceptually essential dimensions for image categorisation. We could arrive at better image groupings and features by studying in which images the distortion discrimination is easier or harder. The results of our model fits can be a starting point for such an analysis. Because better-fitting models correspond more to human performance, images that tend to have relatively high or low values in these metrics can provide insights into which content actually matters. With a regrouping of the images we might be able to find even larger differences between sets of images and it could be possible to find a feature that encodes the difficulty of this task in a new dimension such that the threshold can be gradually shifted based on this dimension. With more insights into the relevant content, an extension to more diverse natural images and distortions in specific regions or patches would be possible.

This work focuses on analyzing image-computable fixed encoding models to measure how well human discrimination performance can be predicted, and we do find promising results with this approach. However, it is not our intention to minimize the influence of top-down processes or flexible encoding models. Previous research has theorized that models of peripheral vision need to introduce an input dependence (Wallis et al., 2019) or that the underestimation of blur and distortions in the periphery is due to a top-down bias toward sharp and undistorted images (Sharvashidze, Valsecchi, & Schütz, 2024; Valsecchi et al., 2018). It well may be that these processes are what is needed on top of low-level information to fully predict human performance.

#### Performance, complexity and sensitivity of the Portilla Simoncelli metric

Our results suggest that the information included in the parameters of the parametric texture model of Portilla and Simoncelli (2000) covers many features relevant to the peripheral discrimination of Eidolon distortions. Because this model was built to synthesize textures, we would expect it to be good at quantifying changes in textures. It is foreseeable that the Portilla and Simoncelli features are useful for this task, since Eidolon distortions could be considered to *texturize* scenes by breaking up object boundaries and edge information. Aspects of these features have also often provided a good accounting of discrimination performance in a range of other stimuli, including both synthesized images and distorted natural images (e.g., Balas, Nakano, & Rosenholtz, 2009; Wallis & Bex, 2012). This is consistent with our finding that the regression model using the Portilla and Simoncelli features performs closer to the experiment-based model than the other metric-based models. However, one should keep in mind that the model comparison with WAIC only measures the complexity (number of effective parameters) of the hierarchical regression model, where the metric is condensed to a single score with a single weight. The model comparison therefore does not take into account the complexity of the metric generation processes themselves. Future work could seek to isolate the model parameters most important for prediction performance (see e.g., Balas, 2006).

In addition, it is noteworthy that the smallest and largest changes measured with the Portilla and Simoncelli (2000) metric occur in texture images (this tendency can be seen in the bluish tails of the data in the first panel of Figure 6). This result could be considered as additional evidence of a limitation of fixed encoding strategies: changes in scene-like images are not captured as well. A metric that could flexibly weight the importance of various features depending on the global organization of the image may perform better across a variety of images.

#### Performance and suitability of other metrics

The low performance of the LGN metrics in the model comparison might be explained by the low sensitivity of the statistics to distortions of small reach (first column in Figure 5) which is similarly present for the metrics only based on spectral information (second column in Figure 5). This low sensitivity might be due to our choice of image distortion, as the distortion’s grain enforces a pattern of a certain spatial frequency regardless of the original input image. This could explain why especially the metric of SC cannot distinguish between images for low reach values. Still, although other metrics perform better they might conceptually make less sense. A metric that fits human perception should show small differences for trials that are hard and large differences for easy trials. However, for example, the JOD (Mantiuk et al., 2021) produces slightly larger differences for textures than for scenes which would be inverse to what we expect from a fitting metric. Therefore, even though the model fit is quite good, it is debatable whether the metric is suitable to encode difficulty in the given task. Extending this analysis to more metrics that might cover relevant features will give more insights into the effect of distortions and their perception.

One limitation of our study is that we did not include a number of image statistics and image-computable vision metrics that would be interesting to evaluate in this setting, such as the Freeman and Simoncelli (2011) model and extensions by Broderick et al. (2023), the texture tiling model (Balas, Nakano, & Rosenholtz, 2009), and other image quality metrics such as SSIM (Wang, Simoncelli, & Bovik, 2003; Wang et al., 2004), LPIPS (Zhang et al., 2018), and DISTS (Ding, Ma, Wang, & Simoncelli, 2020). These omissions were made for a combination of practical reasons, such as code availability and compatibility with our analysis pipeline, required computation time, and our desire to focus first on relatively simple metrics. Because we make our data and code available, others can evaluate metrics of their choice against our data.

#### Change invariance and sensitivity in peripheral vision

Another limitation of this study is that we only tested one class of distortions (Eidolons; Koenderink et al., 2017). Although our results add to the availability of psychophysical data for measuring invariances in peripheral vision, we cannot make statements about the generality of our specific findings to other types of distortions. Regarding invariance to different changes we see a combination of model-free (distortions that do not depend on assuming a specific encoding model of the visual system, such as in (Bex, 2010; Koenderink et al., 2017; Stojanoski & Cusack, 2014; To et al., 2010; Wallis & Bex, 2012) and model-based distortions (in which distorted images are synthesised from a specific encoding model to test some objective (Berardino, Ballé, Laparra, & Simoncelli, 2018; Broderick et al., 2023; Duong et al., 2023; Freeman & Simoncelli, 2011; Zhou, Chun, Subramanian, & Simoncelli, 2023) to be a productive path forward. Model-based distortions allow targeted experimental evaluations of predictions derived from specific models or to compare different models, whereas model-free distortions facilitate model comparison without reference to the correctness or completeness of a specific encoding model. Good models of encoding processes should nevertheless perform well at predicting the discriminability of model-free distortions.

## Figures and Tables

**Figure A1.**
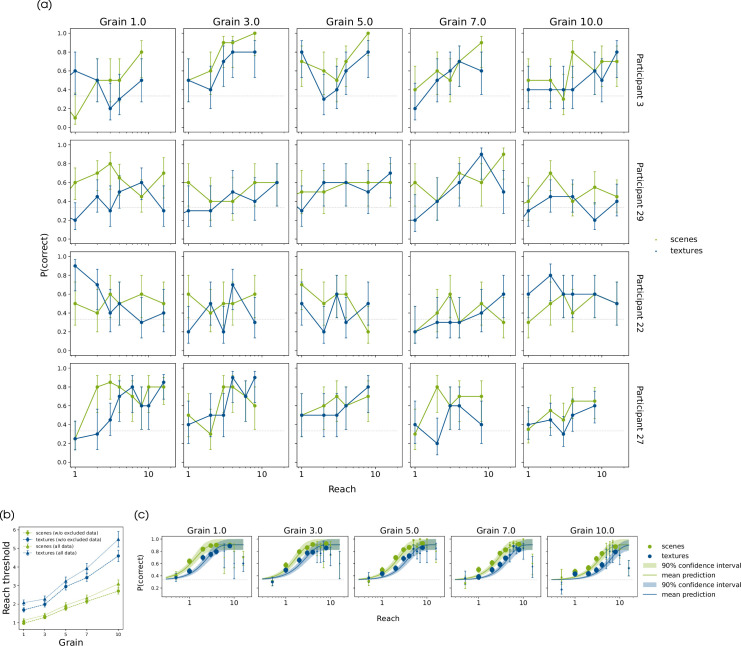
(a) Performance of excluded participants by grain and image condition. (b) Posterior estimate for thresholds in the main experiment by grain and image condition based as shown in Figure 4b and when using all data without any exclusion. Error bars indicate the standard deviation of the posterior distribution. (c) Fitted functions for data in the main experiment by image condition, and grain based on all data without any exclusion. Data points show the mean performance for the pooled data over the corresponding trials. The size of the data points indicate the amount of pooled data, i. e. the larger the point the more data is included. Error bars on the data points show the 90% credible interval of the beta distribution estimate.

## Footnotes

1 In the Eidolon Factory this is implemented in the disarray_image function.

2 We acknowledge that some of the image-computable quantities considered here are not metrics in their mathematical definition, but measures that might be used to quantify image difference.

## Appendix

### LGN algorithm

The algorithm to derive spatial coherence (*SC*) and contrast energy (*CE*) given in Groen et al. (2013) includes the following four steps:

(1) **Color transformation**: Each image was transformed from RGB to an opponent color model (Koenderink et al., 1972) with luminance following ITU-R (2011) and absolute channel weights summing to 1:
  $$\begin{array}{c} I=\left[\begin{array}{c} L\\ Y-B\\ R-G \end{array}\right]=\left[\begin{array}{ccc} 0.3 & 0.58 & 0.11\\ 0.25 & 0.25 & -0.5\\ 0.5 & -0.5 & 0 \end{array}\right]\cdot \left[\begin{array}{c} R\\ G\\ B \end{array}\right] \end{array}$$
(2) **Receptive field responses**: We modeled responses of receptive fields with varying sizes by performing the next steps with varying standard deviations σ. For CE we used σ ∈ {3, 6, 12, 24, 48} and for SC σ ∈ {4, 8, 16, 32, 64}. We then computed the response as follows:
  (a) One-dimensional zero-mean Gaussian filters with size of 3 standard deviations σ were applied horizontally *G*_*x*_ and vertically *G*_*y*_ to each color channel separately.
  (b) The filter responses were combined as a sum of squares into one response array:
    $$\begin{array}{c} R\left(I;\sigma \right)=\sqrt{G_{x}{\left(I;\sigma \right)}^{2}+G_{y}{\left(I;\sigma \right)}^{2}} \end{array}$$
(3) To normalize the response we computed the local coefficient of variation *CV*:
  $$\begin{array}{c} CV\left(I;\sigma \right)=\frac{SD(I;\sigma )}{E(I;\sigma )} \end{array}$$
  with
  $$\begin{array}{c} E\left(I;\sigma \right)=G_{xy}\left(R\left(I;\sigma \right);\sigma \right) \end{array}$$
  and
  $$\begin{array}{ccc} & & \;SD(I;\sigma )\\ & & \;=\sqrt{\max[G_{xy}\left(R{\left(I;\sigma \right)}^{2};\sigma \right)-G_{xy}{\left(R\left(I;\sigma \right);\sigma \right)}^{2},0]}, \end{array}$$
  where *G*_*xy*_ is the response from a two-dimensional zero-mean Gaussian filter. With this local coefficient of variation, the response was normalized by:
  $$\begin{array}{c} \bar{R}\left(I;\sigma \right)=\frac{R(I;\sigma )\cdot \max(R(I;\sigma ))}{R(I;\sigma )+\max(R(I;\sigma ))\cdot CV(I;\sigma )}. \end{array}$$
(3) **Minimum reliable scale selection**: For each set of standard deviations the responses were combined by taking the above-threshold filter activation with the smallest σ for each pixel:
  $$\begin{array}{ccc} \sigma^{*}\left(x,y\right) & \;= & \min\left\{\sigma |\bar{R}\left(I;\sigma \right)\left(x,y\right)-t_{\sigma }>ɛ\right\}\\ R_{LGN}\left(x,y\right) & \;= & \bar{R}\left(I;\sigma^{*}\left(x,y\right)\right)\left(x,y\right). \end{array}$$
  Consequently, this step produces one edge map of the image for each of the two sets of standard deviations.
(4) **Pooling**: To derive scalar scores we averaged the activation over a centered circle with radius *r*_*CE*_ = 75 px and *r*_*SC*_ = 250 px, respectively. The two output values were then the means of these pooled activations over the three color channels.

### Eidolon distortion algorithm

For a given image *I* we generated the Eidolon distortion (Koenderink et al., 2017) with grain *g* and reach *r* as follows.

(1) **Grain-size Gaussian**. We start with a two-dimensional Gaussian filter kernel with standard deviation *grain* *g* with the size of the image:
  $$\begin{array}{c} k\left(x,y\right)=e^{-\frac{x^{2}+y^{2}}{2g^{2}}}. \end{array}$$
(2) **Gaussian vector field**. We sampled two Gaussian matrices of the same size from
  $$G_{x}\left(x,y\right),G_{y}\left(x,y\right)∼N\left(0,1\right)\;\forall \;x,y.$$
  We create the *x* and *y* component of the random vector field by element-wise multiplying the kernel with each of the matrices in frequency space and reversing the Fourier Transformation F to get a vector field in signal space:
  $$\begin{array}{ccc} v_{x} & \;= & F^{-1}\left(F\left(k\right)⊙F\left(G_{x}\right)\right),\\ v_{y} & \;= & F^{-1}\left(F\left(k\right)⊙F\left(G_{y}\right)\right). \end{array}$$
(3) **Disarray pixels**. The distorted image is generated by shifting its pixels by the normalized vector field weighted with *reach* *r*:
  $$\begin{array}{c} \;\bar{I}\left(x,y\right)=I\left(x+r\cdot \frac{v_{x}\left(x,y\right)}{∥v_{x}∥},y+r\cdot \frac{v_{y}\left(x,y\right)}{∥v_{y}∥}\right). \end{array}$$
  To avoid mixing the colors, the same disarray was applied to each *RGB*-color channel.

### Excluded data

During data collection we realized that some of the participants didn’t improve in performance even for very strong distortions. This prompted us to set an exclusion criterion as we suspect these participants weren’t performing the task correctly. We therefore excluded all participants with an overall performance below 60% form further data analysis. This exclusion criterion resulted in the exclusion of four participants. The aggregated data by reach of these four participants is shown in Figure A1a per participant, image condition, and grain.

For comparison with the results presented in the paper, Figures A1b and A1c show the model fits and parameters on all data without excluding any participants or trials. We can see that the primary difference of interest (reach thresholds between scene and texture images) remain robust. Qualitatively the data patterns are similar to the results shown in Figures 4a and 4b, with reach thresholds simply being higher when computed on all data. We opted to perform the metric-based analysis on the reduced data set, to make the results of this analysis more representative of the behavior of participants who could perform the task well.

### Metrics

#### Change of mean color

For the simplest measure of change, we consider the difference in mean color which holds no information about spatial changes:

$$\begin{array}{ccc} d_{RG}\left(I,\bar{I}\right) & \;= & \frac{1}{n^{2}}\sum_{x,y}^{n}\bar{RG}\left(x,y\right)-\frac{1}{n^{2}}\sum_{x,y}^{n}RG\left(x,y\right),\\ d_{BY}\left(I,\bar{I}\right) & \;= & \frac{1}{n^{2}}\sum_{x,y}^{n}\bar{BY}\left(x,y\right)-\frac{1}{n^{2}}\sum_{x,y}^{n}BY\left(x,y\right). \end{array}$$

#### Mean squared error

The mere physical difference between the images is measured by the mean squared difference between the pixels of the original and the distorted image:

$$\begin{array}{c} MSE\left(I,\bar{I}\right)=\frac{1}{n^{2}}\sum_{x,y}^{n}{\left(I\left(x,y\right)-\bar{I}\left(x,y\right)\right)}^{2} \end{array}$$

#### Change of spectral information

To integrate more spatial information we also considered three difference measures based on the discrete frequency spectrum of the gray channel (F(G)). Firstly, the Manhattan metric on the rotational mean *r* of the spectrum,

$$\begin{array}{c} d_{\text{spectrum}}\left(I,\bar{I}\right)=\sum_{x,y}^{n}\left|r\left(F\left(\bar{G}\right)\right)-r\left(F\left(G\right)\right)\right|, \end{array}$$

which approximates the integral of the difference between the two functions. Secondly, the intercept (*FI*) and slope (*FS*) of a linear function fitted to said rotational mean.

#### Change in LGN statistics

Moving towards more biologically inspired measures we analyzed the change in the LGN statistics through their difference

$$\begin{array}{ccc} d_{CE}\left(I,\bar{I}\right) & \;= & CE\left(\bar{I}\right)-CE\left(I\right),\\ d_{SC}\left(I,\bar{I}\right) & \;= & SC\left(\bar{I}\right)-SC\left(I\right), \end{array}$$

and their combined Euclidean distance:

$$\begin{array}{c} d_{LGN}=\sqrt{d_{CE}{\left(I,\bar{I}\right)}^{2}+d_{SC}{\left(I,\bar{I}\right)}^{2}}. \end{array}$$

#### Change in texture parameters

The parametric texture model by Portilla and Simoncelli (2000) uses a multi-scale wavelet representation with 2461 parameters to capture marginal statistics of pixel intensities, distributions of spatial frequencies as well as many correlation measures between those values, all across high- and low-pass bands and different scales. Those parameters can be fitted to an image and then used to resynthesize a new image which will have similar properties in texture. Consequently, the fitted parameters contain some information about the images’ spatial regularities and features. We computed this using the plenoptic (Duong et al., 2023) package in python and built the model with the default parameters (the same as in Portilla & Simoncelli, 2000). To fulfill the constraint of the model that the image size must be divisible by 2^*N*^ (*N* is the number of scales in the image pyramid, default is *N* = 4), we padded our images with reflect-mode to increase their size from 350 × 350 to 352 × 352. Here we define two difference measures by determining the Euclidean distance as well as the Cosine similarity of the parameters fitted to the original *PS*(*I*) and the distorted image $PS(\bar{I})$:

$$\begin{array}{ccc} d_{PS;\text{Euclid}}\left(I,\bar{I}\right) & \;= & ∥PS\left(I\right)-PS\left(\bar{I}\right)∥,\\ d_{PS;\cos}\left(I,\bar{I}\right) & \;= & \frac{PS\left(I\right)\cdot PS\left(\bar{I}\right)}{∥PS\left(I\right)∥\cdot ∥PS\left(\bar{I}\right)∥}. \end{array}$$

We expect the Euclidean distance to work well for our use case because its actually the error that is minimized in the metamer synthesis process the model was built for Portilla and Simoncelli (2000).

#### Quality (just-objectionable-difference) based on FovVideoVDP

Lastly, the model by Mantiuk et al. (2021) named FovVideoVDP derives a (video) difference metric based on spatial, and peripheral aspects of perception. This metric is measured in just-objectionable-difference (JOD) units where 10 means no difference and the measure decreases with larger image differences.

#### Normalized versus non-normalized difference

To cover the case that the change relative to the image’s original value is more informative for each measure based on a single difference we additionally computed a normalized metric by dividing the difference by the corresponding statistical value of the original image. For the Euclidean and Manhattan metric, we analogously normalized the results with the Euclidean (ℓ_2_) and ℓ_1_ norm of the values for the original image, respectively.
